# Interceptive Treatment of Class II Malocclusion in Pediatric Patients Using Clear Aligner Mandibular Advancement: A Systematic Review Following PRISMA Guidelines

**DOI:** 10.7759/cureus.82089

**Published:** 2025-04-11

**Authors:** Ahmed S Khalil, Rawan S Alrehaili, Zainab Mahmoud, Rasha Alrashidi, Anwar Alkhalaf, Mohammed Abdelmaksoud, Loulwah Linjawi, Reem Alsahafi, Zainab Alghareeb, Fahad Bujbarah, Nada Shahir, Ali A Assiry

**Affiliations:** 1 Orthodontics, Private Practice, Medina, SAU; 2 Dentistry, Private Practice, Medina, SAU; 3 College of Dentistry, Vision College, Riyadh, SAU; 4 College of Dentistry, King Abdulaziz University, Jeddah, SAU; 5 College of Dentistry, Imam Abdulrahman Bin Faisal University, Dammam, SAU; 6 College of Dentistry, King Khalid University, Abha, SAU; 7 Preventive Dental Science, Faculty of Dentistry, Najran University, Najran, SAU

**Keywords:** aligner mandibular advancement, class ii malocclusion, clear aligners, functional appliance, interceptive treatment, invisalign, invisalign mandibular advancement, pediatric patients

## Abstract

While conventional functional appliances have been widely used to manage Class II malocclusion in growing pediatric patients, advancements in interceptive treatments have introduced aligner mandibular advancement as a modern and aesthetic alternative. Despite its increasing popularity, the effectiveness of aligner mandibular advancement has not been systematically evaluated. This systematic review aimed to evaluate the effectiveness of interceptive aligner mandibular advancement in the treatment of Class II malocclusion in pediatric patients, focusing on skeletal, dental, and soft tissue outcomes.

This review adhered to the Preferred Reporting Items for Systematic reviews and Meta-Analyses or PRISMA guidelines. Studies evaluating pediatric patients with Class II malocclusion treated with aligner mandibular advancement were included. The databases searched included PubMed, Cochrane Library, Web of Science, Embase, and Scopus, with searches conducted up to November 30, 2024, to identify relevant articles addressing the PICOS framework (Population, Intervention, Comparison, Outcome, and Study design). The focus questions were: How effective is clear aligner mandibular advancement in the treatment of pediatric patients with Class II malocclusion? What are its associated treatment outcomes? The ROBINS-I tool was used to assess the risk of bias. Data were synthesized narratively due to heterogeneity in study designs and outcomes. Nine studies with 426 participants were included, conducted between 2021 and 2024. Seven studies adopted retrospective designs, one study used a prospective design, and one relied on a survey-based design. Aligner mandibular advancement demonstrated effectiveness in reducing overjet and ANB while maintaining lower incisor inclination compared to conventional functional appliances. Soft tissue improvements were also reported. Patients favored aligner mandibular advancement over conventional functional appliances for its superior comfort, better aesthetics, and less breakage. However, most studies were retrospective, and the lack of randomized controlled trials limited the strength of the evidence.

Clear aligner mandibular advancement appears to be an effective approach for managing Class II malocclusions in growing pediatric patients, offering the distinct advantage of maintaining lower incisor inclination compared to other conventional functional appliances. However, the evidence remains limited due to methodological constraints, including the lack of randomized controlled trials, small sample sizes, and inconsistencies in study designs.

## Introduction and background

Class II malocclusion is characterized by a protruded maxilla, retruded mandible, or a combination of both [1-4]. It is typically managed using one of three primary approaches: modifying growth patterns, orthodontic camouflage with or without extraction, or orthognathic surgery [5]. Growth modification often involves a two-stage treatment process, with the first stage focusing on altering growth patterns and the second stage addressing any remaining occlusal discrepancies [6-8]. Growth modification frequently uses headgear, which redirects maxillary growth while promoting forward mandibular development [9-11]. Functional appliances are a widely favored treatment choice for addressing Class II, division 1 malocclusion, particularly in cases associated with mandibular retrusion [12]. These appliances are specifically designed to address dentoskeletal discrepancies and help achieve better occlusal harmony [13-16]. Functional appliances work by repositioning the mandibular condyle in a more forward position within the glenoid fossa, hence stimulating mandibular growth [17]. In addition to skeletal changes, these appliances also exert dentoalveolar effects, typically resulting in retroclination of upper incisors and proclination of lower incisors [18-20].

Recently, the scope of clear aligner therapy has remarkably expanded, progressing from addressing simpler cases to more complex treatments, including malocclusions requiring orthognathic surgery, extractions, and functional appliances for Class II correction in growing patients [21-26]. Invisalign with mandibular advancement is a novel appliance that integrates clear aligner technology with functional mechanics. It features a series of clear aligners customized for each dental arch and equipped with precision wings, which are lateral inclined planes designed to guide the mandible into a forward position [27]. This design allows for incremental tooth movement while mimicking the other tooth-borne removable functional appliances used for growth modification in patients with Class II malocclusions.

While limited research exists on this topic, no published article has systematically analyzed the effectiveness of aligner mandibular advancement in growing patients with Class II malocclusion. Understanding its effectiveness and clinical outcomes will allow clinicians to make evidence-based decisions, optimize treatment protocols, and better address the unique needs of pediatric patients requiring interceptive treatment. Therefore, the aim of the current systematic review is to evaluate the effectiveness of aligner mandibular advancement in pediatric patients and to assess its associated skeletal, dental, and soft tissue treatment effects.

## Review

Methods

Eligibility Criteria

The current systematic review was performed in accordance with the Preferred Reporting Items for Systematic Reviews and Meta-Analysis (PRISMA) guidelines [28] and was registered to the Open Science Framework database (osf.io/2m3kg). The objective of this review was to address the focus questions: How effective is clear aligner mandibular advancement in the treatment of pediatric patients with Class II malocclusion? What are its associated treatment outcomes? The selection criteria were applied using the PICOS framework, which refers to Population, Intervention, Comparison, Outcome, and Study design, as follows:

Population: pediatric patients with Class II malocclusion

Intervention: interceptive treatment with clear aligner mandibular advancement

Comparison: conventional functional appliances

Outcome: the efficiency of clear aligner mandibular advancement in interceptive treatment of pediatric patients with Class II malocclusion was the primary outcome, while the treatment effects achieved served as the secondary outcome.

Study design: prospective, retrospective, and observational studies were considered eligible for inclusion in this review.

The studies included in this review focused on the outcomes of clear aligner mandibular advancement in pediatric patients. Only studies published in English were deemed eligible for inclusion. Conversely, research involving animal studies, case reports, case series, review articles, systematic reviews, meta-analyses, editorials, and commentaries were excluded. Furthermore, studies focusing on patients needing orthognathic surgery or those published in languages other than English were not considered.

Information Sources and Search Strategy

A thorough electronic search was conducted across multiple databases, including PubMed, Cochrane Library, Web of Science, Embase, and Scopus, to identify relevant studies published from 2017 to November 30, 2024. The primary aim of the search was to assess the efficiency of clear aligner mandibular advancement in the interceptive treatment of pediatric patients with Class II malocclusion, as well as to analyze the associated treatment effects. Additionally, reference lists of the included studies were manually reviewed to identify any supplementary relevant articles.

The search strategy in PubMed used a combination of keywords and MeSH terms to ensure a comprehensive retrieval of studies. The syntax included terms such as: (("clear aligners" OR "Invisalign" OR "orthodontic aligners" OR "aligner therapy") AND ("class II malocclusion" OR "mandibular advancement" OR "functional appliances" OR "malocclusion, "Angle Class II"[MeSH Terms]) AND ("interceptive orthodontics" OR "orthodontic treatment" OR "pediatric treatment" OR "orthodontics, corrective"[MeSH Terms]) AND ("treatment effects" OR "mandibular growth" OR "bite correction")). The search syntax was adapted for each database to align with its respective indexing system and search requirements.

Study Selection and Assessment

Two researchers independently conducted the initial screening of titles and abstracts. Subsequently, the full-text articles were thoroughly reviewed to verify their eligibility for inclusion. Any uncertainties or disagreements encountered during this process were addressed and resolved through discussion.

Data Extraction

Two researchers independently carried out data extraction, and any differences were resolved through discussion to ensure consensus. This careful process guaranteed that only studies directly relevant to the research question were included, thereby strengthening the review's reliability and validity. The extracted data from the studies included: demographic details (country, gender distribution, participants' mean age, and sample size), study design, intervention performed, treatment duration, methods of assessment, comparison groups, changes in overjet and overbite, skeletal and dental changes, soft tissue changes, and key findings.

Quality Assessment

The methodological quality of the selected studies was independently assessed by two researchers using the ROBINS-I tool [29]. The evaluation of bias covered seven key domains: confounding, selection bias, intervention classification, deviations from intended interventions, missing data, outcome measurement, and selection of reported results. Each domain was reviewed individually, culminating in an overall assessment of the risk of bias, which was classified as low, moderate, serious, critical, or insufficient information. Any disagreements between the reviewers’ assessments were addressed and resolved through discussion to achieve consensus.

Results

Study Selection

**Figure 1:**
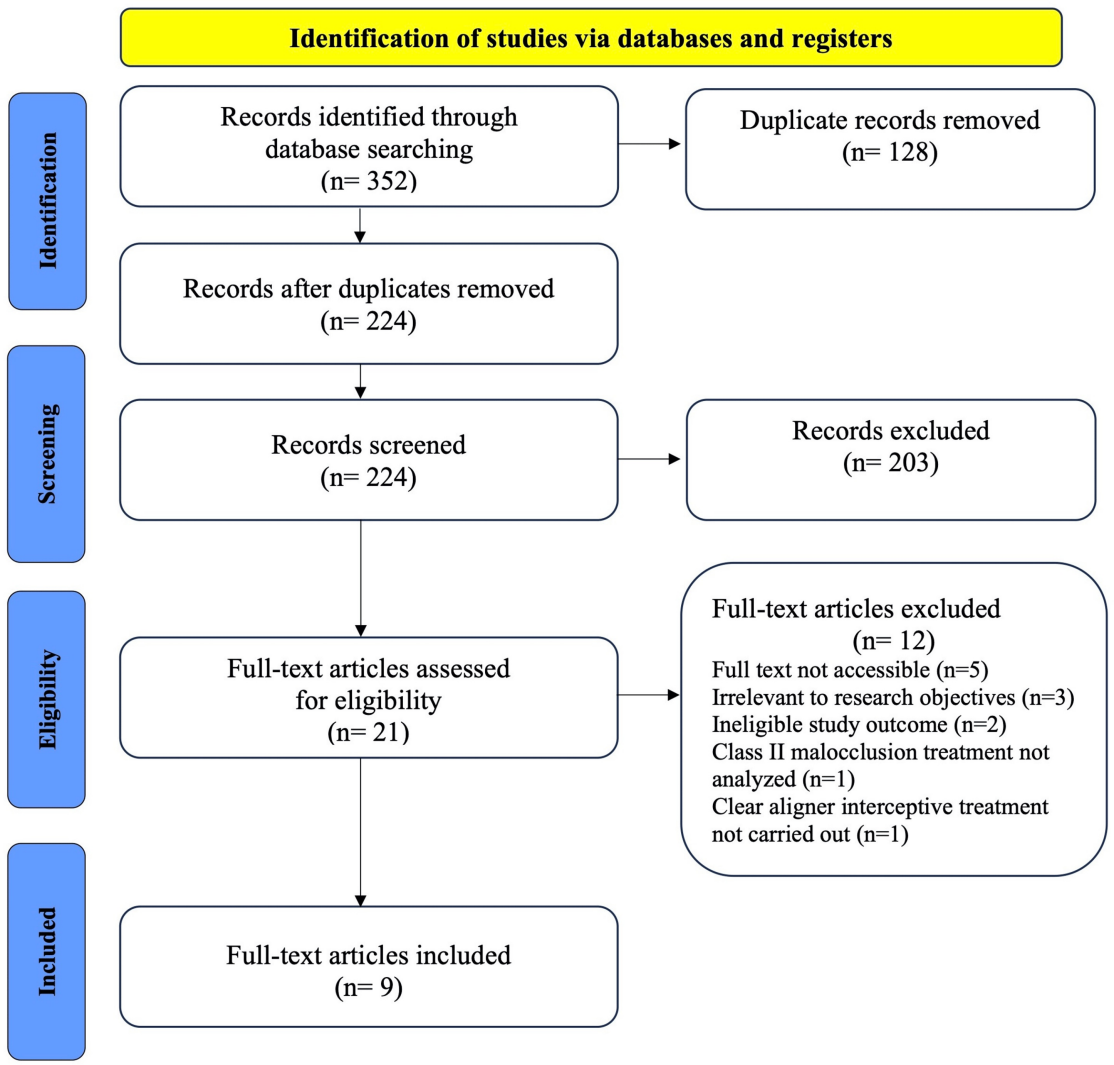
PRISMA flowchart depicting the study selection process PRIMA, Preferred Reporting Items for Systematic Reviews and Meta-Analysis

A total of 352 records were identified through database searches. After removing duplicates, 224 records were retained for initial screening based on titles and abstracts. Of these, 203 records were excluded due to irrelevance or duplication, leaving 21 articles for full-text review. During the eligibility assessment, 12 articles were excluded for the following reasons: five articles had inaccessible full texts, three were irrelevant to the research objectives, two had ineligible study outcomes, one did not analyze Class II malocclusion treatment, and one did not involve clear aligner interceptive treatment. Finally, nine studies meeting the inclusion criteria were selected for the systematic review [30-38]. These studies provided comprehensive insights into the use of aligner mandibular advancement in interceptive treatment for pediatric patients with Class II malocclusion. The search process following the PRISMA flow methodology is depicted in Figure 1.

Study Characteristics

All nine studies included in this systematic review were conducted between 2021 and 2024, with a total of 426 participants. Seven studies adopted retrospective design [30,33-38], one used a prospective design [31], and one relied on a survey-based design [32]. The included studies were conducted in various countries, including Italy, Canada, China, the United States, and the United Arab Emirates. Some studies were conducted in university settings [30,34,38], while three studies used private practice records or multicenter designs [31,33,36]. Sample sizes varied across studies, ranging from 15 participants [33] to 71 participants [37].

Gender distribution was generally balanced across most studies, with some showing an equal number of male and female participants [30,37,38], while others reported a slight predominance of females [35,36]. However, one study did not specify gender distribution [31]. Mean age across studies ranged between 10 [30] and 13 years [36] depending on the treatment group (Table 1).

**Table 1:** Demographic data of the included studies CVM, cervical vertebral maturation; SD, standard deviation

Authors and Year	Location	Study Setting	Sample Size	Gender Distribution	Mean Age in Years (SD)
Caruso et al., 2021 [30]	Italy	University setting	N=20 (10 aligner mandibular advancement, 10 Twin Block)	10 males, 10 females	10 (±1.03)
Ravera et al., 2021 [31]	Italy	Private practice	N=68 (40 Invisalign, 28 control)	Not reported	CVM2: 9.2 (±1.4); CVM3: 12.10 (±1.4)
Zybutz et al., 2021 [32]	Canada	University setting and private practice	N=68 (45 Invisalign teen, 23 Twin Block)	Invisalign: 18 boys, 27 girls; Twin Block: 13 boys, 10 girls	Invisalign: 13.62 (±1.54); Twin Block: 10.60 (±1.92)
Awad and Sadek, 2022 [33]	United Arab Emirates	Private practice	N=15 (all treated with aligner mandibular advancement)	10 females, 5 males	11.6 (±2.35)
Wu et al., 2023 [34]	China	University setting	N=63 (14 aligner mandibular advancement, 12 control, 14 van Beek activator, 11 Herbst, 12 Twin Block)	Aligner mandibular advancement: 12 boys, 2 girls; control: 7 boys, 5 girls; van Beek activator: 7 boys, 7 girls; Herbst: 4 boys, 7 girls; Twin Block: 7 boys, 5 girls	Aligner mandibular advancement: 12.11 (±1.16); control: 10.41 (±0.90); van Beek activator: 10.71 (±1.44); Herbst: 11.55 (±0.69); Twin Block: 11.00 (±1.04)
Yue et al., 2023 [35]	China	University setting	N=32 (16 aligner mandibular advancement, 16 Twin Block)	Aligner mandibular advancement: 7 boys, 9 girls; Twin Block: 8 boys, 8 girls	Aligner mandibular advancement: 10.02 (±0.99); Twin Block: 10.38 (±0.68)
Hosseini et al., 2024 [36]	United States and Canada	University setting and private practice	N=40 (20 Invisalign, 20 Herbst)	Invisalign: 9 males, 11 females; Herbst: 9 males, 11 females	Invisalign: 13.1 (±1.5); Herbst: 12.7 (±1.8)
Lombardo et al., 2024 [37]	Italy	University setting	N=71 (35 Twin Block, 21 aligner mandibular advancement, 15 control)	Twin Block: 17 males, 18 females; aligner mandibular advancement: 9 males, 12 females; control: 4 males, 11 females	Twin Block: 12.0 (±1.3); aligner mandibular advancement: 11.2 (±1.1); control: 10.9 (±1.1)
Zhang et al., 2024 [38]	China	University setting	N=49 (24 Twin Block, 25 aligner mandibular advancement)	Twin Block: 12 males, 12 females; aligner mandibular advancement: 12 males, 13 females	Twin Block: 10.71 (±0.86); aligner mandibular advancement: 10.96 (±0.84)

The included studies primarily focused on growing patients with skeletal Class II malocclusions due to mandibular retrusion. Most studies compared aligner mandibular advancement to the Twin Block appliance [30,32,34,35,37,38], while two studies evaluated aligner mandibular advancement against the Herbst appliance [34,36]. Other studies included untreated control groups [31] or focused solely on aligner mandibular advancement [33]. Treatment durations varied across the studies. Assessment methods primarily involved lateral cephalometric radiographs in most studies, while two studies incorporated cone beam computed tomography (CBCT) imaging [35,38]. Initial overjet values ranged from 5 mm to 7.2 mm (Tables 2, 3).

**Table 2:** Study characteristics of the included studies CBCT, cone beam computed tomography; CVM, cervical vertebral maturation; NS, not specified

Authors and Year	Study Design	Average Treatment Duration (Months)	Intervention	Assessment Method	Comparison
Caruso et al., 2021 [30]	Retrospective controlled study	NS	Aligner mandibular advancement, Twin Block	Lateral cephalometric radiographs	Aligner mandibular advancement vs. Twin Block
Ravera et al., 2021 [31]	Prospective controlled study	CVM2: 18, CVM3: 17	Invisalign mandibular advancement divided into two subgroups with regard to the growth stage	Lateral cephalometric radiographs	Mandibular advancement at CVM2 and CVM3 vs. controls
Zybutz et al., 2021 [32]	Survey-based study	NS	Invisalign teen with mandibular advancement, Twin Block	Survey-based assessments	Invisalign teen mandibular advancement vs. Twin Block
Awad and Sadek, 2022 [33]	Retrospective study	17.73	Invisalign mandibular advancement	Lateral cephalometric radiographs	No control group
Wu et al., 2023 [34]	Retrospective comparative study	Mandibular advancement: 22.84, van Beek activator: 7.28, Herbst: 10.18, Twin Block: 10.16	Mandibular advancement, van Beek activator, Herbst, Twin Block	Lateral cephalometric radiographs	Mandibular advancement, van Beek activator, Herbst, Twin Block vs. controls
Yue et al., 2023 [35]	Retrospective comparative study	Mandibular advancement: 11.45, Twin Block: 12.11	Invisalign mandibular advancement, Twin Block	CBCT, lateral cephalometric radiographs	Invisalign mandibular advancement vs. Twin Block
Hosseini et al., 2024 [36]	Retrospective comparative study	Mandibular advancement: 32.3, Herbst: 36.8	Invisalign mandibular advancement, Herbst	Lateral cephalometric radiographs	Mandibular advancement vs. Herbst
Lombardo et al., 2024 [37]	Retrospective controlled study	NS	Invisalign mandibular advancement, Twin Block	Lateral cephalometric radiographs	Mandibular advancement vs. Twin Block
Zhang et al., 2024 [38]	Retrospective study	Mandibular advancement: 11.82, Twin Block: 12.15	Aligner mandibular advancement, Twin Block	CBCT	Mandibular advancement vs. Twin Block

**Table 3:** Measured changes and results of the included studies CVM, cervical vertebral maturation; NS, not specified; SD, standard deviation

Authors and Year	Change in Overjet (Mean ± SD, mm)	Change in Overbite (Mean ± SD, mm)	Skeletal Changes			Dental Changes		Soft Tissue Changes	Results
			Change in ANB (Mean ± SD, Degrees)	Change in SNA (Mean ± SD, Degrees)	Change in SNB (Mean ± SD, Degrees)	Change in Upper Incisors	Change in Lower Incisors		
Caruso et al., 2021 [30]	Aligner mandibular advancement: -1.4, Twin Block: -3.3	Aligner mandibular advancement: -3.15, Twin Block: -0.1,	Aligner mandibular advancement: -3.4, Twin Block: -5.6	Aligner mandibular advancement: +0.2, Twin Block: -3,	Twin Block: +3.2, aligner mandibular advancement: +4.4	Aligner mandibular advancement maintained upper incisor angulation while the Twin Block caused retroclination of upper incisors by 10°.	Aligner mandibular advancement resulted in proclination by 2.4° while the Twin Block caused slight retroclination of lower incisors by 1.5°.	NS	The aligner mandibular advancement provided better control over upper and lower incisors.
Ravera et al., 2021 [31]	CVM2: -2.59 CVM3: NS	NS	CVM2: -1.30 CVM3: -0.91	No significant change	No significant change	Aligner mandibular advancement caused retroclination of upper incisors by 6.05°.	There was no significant change in lower incisor inclination.	NS	Aligner mandibular advancement produced dentoalveolar changes at CVM2 and skeletal effects at CVM3 (Wits appraisal decreased by -3.65 mm).
Zybutz et al., 2021 [32]	NS							Patients using aligner mandibular advancement reported higher lip soreness compared to those using the Twin Block.	The aligner mandibular advancement was better in terms of patient comfort and resulted in fewer appliance breakages compared to the Twin Block.
Awad and Sadek, 2022 [33]	-3.80	+0.07	-1.53	+0.59	+2.14	Aligner mandibular advancement caused retroclination of upper incisors by 7.03°.	There was no significant change in lower incisor inclination.	The aligner mandibular advancement improved facial convexity and reduced upper lip protrusion.	The aligner mandibular advancement effectively reduced overjet and improved facial convexity, providing good control of upper and lower incisors.
Wu et al., 2023 [34]	Aligner mandibular advancement: -3.66, van Beek activator: -2.77, Herbst: -5.53, Twin Block: -4.73	NS	Aligner mandibular advancement: -0.85, van Beek activator: -1.00, Herbst: -1.80, Twin Block: -1.10	Aligner mandibular advancement: +0.18, other appliances showed no significant change.	Aligner mandibular advancement: +0.90, van Beek activator: +0.90, Herbst: +1.50, Twin Block: +1.10	The aligner mandibular advancement maintained upper incisor angulation with minimal changes, while other appliances caused retroclination of 5°–10°.	The aligner mandibular advancement showed slight control over lower incisor angulation with minimal proclination, whereas other appliances caused proclination by 5°–8°.	NS	The aligner mandibular advancement effectively treated Class II malocclusion and provided better control of incisors compared to the van Beek activator, Herbst, and Twin Block appliances.
Yue et al., 2023 [35]	Both groups achieved normal overjet and overbite after treatment, but specific changes were not quantified.		Significant reduction	No significant change	Significant improvement	NS			Both aligner mandibular advancement and the Twin Block expanded the airway, but aligner mandibular advancement showed better improvement in the hypopharynx.
Hosseini et al., 2024 [36]	Aligner mandibular advancement: -2.8, Herbst: -4.8	Aligner mandibular advancement: -1.4, Herbst: -3.3	Aligner mandibular advancement: -1.5, Herbst: -1.8	Aligner mandibular advancement: -0.8, Herbst: -1.2	Aligner mandibular advancement: +0.7, Herbst: +0.5	Aligner mandibular advancement caused retroclination of upper incisors by 2.2°, while the Herbst appliance caused retroclination by 6.8°.	Aligner mandibular advancement caused retroclination of lower incisors by 2.1°, while the Herbst appliance caused proclination by 7.4°.	The aligner mandibular advancement improved the soft tissue profile with better vertical control, while the Herbst appliance increased facial height.	The aligner mandibular advancement was effective in dolichofacial cases, while the Herbst appliance was more effective in correcting overbite.
Lombardo et al., 2024 [37]	Twin Block: -3.7, aligner mandibular advancement: -2.0	Twin Block: -2.6, aligner mandibular advancement: -1.8	Twin Block: -1.5, aligner mandibular advancement: -1.5	No significant changes	Twin Block: +1.6, aligner mandibular advancement: +1.4	Both the Twin Block and aligner mandibular advancement provided effective control of upper incisor inclination.	Neither the Twin Block nor aligner mandibular advancement caused significant proclination of the lower incisors.	The Twin Block showed greater advancement of the pogonion compared to aligner mandibular advancement.	Both aligner mandibular advancement and the Twin Block effectively treated Class II malocclusion, with the Twin Block showing slightly better improvements in soft tissue profile.
Zhang et al., 2024 [38]	NS								Both aligner mandibular advancement and the Twin Block were effective in remodeling the temporomandibular joint.

Quality Assessment

Overall, all nine studies had a moderate risk of bias as depicted in Table 4. While most studies demonstrated robust methodologies, some limitations were consistently observed across key domains. The primary areas of concern were confounding and selection bias, as none of the studies employed randomization, and potential confounders, such as compliance or environmental factors, were not fully addressed. Retrospective designs used in the majority of the studies further increased the risk of selection bias by potentially favoring patients with complete and successful treatment records. In contrast, the domains of deviations from intended interventions, measurement of outcomes, and selection of reported results generally exhibited low risk of bias. Most studies adhered to standardized treatment protocols, used validated tools such as lateral cephalograms or CBCT, and reported outcomes comprehensively and transparently. However, transparency regarding missing data was limited in all studies, with insufficient reporting on exclusions. This lack of clarity contributed to a moderate risk of bias in this domain across all studies.

**Table 4:** Risk of bias assessment for included studies using ROBINS-I tool

Authors and Year	Bias Due to Confounding	Bias in Selection of Participants	Bias in Classification of Interventions	Bias Due to Deviations from Intended Interventions	Bias Due to Missing Data	Bias in Measurement of Outcomes	Bias in Selection of Reported Results	Overall Risk of Bias
Caruso et al., 2021 [30]	Moderate risk	Moderate risk	Low risk	Low risk	Moderate risk	Low risk	Low risk	Moderate risk
Ravera et al., 2021 [31]	Moderate risk	Moderate risk	Moderate risk	Low risk	Moderate risk	Low risk	Low risk	Moderate risk
Zybutz et al., 2021 [32]	Moderate risk	Moderate risk	Moderate risk	Low risk	Moderate risk	Moderate risk	Low risk	Moderate risk
Awad and Sadek, 2022 [33]	Moderate risk	Moderate risk	Low risk	Low risk	Moderate risk	Low risk	Low risk	Moderate risk
Wu et al., 2023 [34]	Moderate risk	Moderate risk	Moderate risk	Low risk	Moderate risk	Low risk	Low risk	Moderate risk
Yue et al., 2023 [35]	Moderate risk	Moderate risk	Moderate risk	Low risk	Moderate risk	Low risk	Low risk	Moderate risk
Hosseini et al., 2024 [36]	Moderate risk	Moderate risk	Moderate risk	Low risk	Moderate risk	Low risk	Low risk	Moderate risk
Lombardo et al., 2024 [37]	Moderate risk	Moderate risk	Moderate risk	Low risk	Moderate risk	Low risk	Low risk	Moderate risk
Zhang et al., 2024 [38]	Moderate risk	Moderate risk	Moderate risk	Low risk	Moderate risk	Low risk	Low risk	Moderate risk

Results of Individual Studies and Main Findings

A quantitative statistical meta-analysis was not conducted due to the considerable heterogeneity in study designs and methodologies. Differences were noted in treatment protocols, patient demographics, and reported outcomes among the included studies. Caruso et al. [30] reported that both aligner mandibular advancement and the Twin Block were effective in correcting Class II malocclusion. Aligner mandibular advancement provided superior control over upper incisors, while the Twin Block demonstrated greater mandibular growth and skeletal improvements. Ravera et al. [31] demonstrated that aligner mandibular advancement produced significant dentoalveolar changes in prepubertal patients (cervical vertebral maturation or CVM2), while pubertal patients (CVM3) experienced more pronounced skeletal effects. The appliance effectively reduced overjet and ANB in both growth stages. Zybutz et al. [32] highlighted that patients preferred aligner mandibular advancement over the Twin Block due to greater comfort, better aesthetics, and fewer appliance breakages. Both appliances were effective in treating Class II malocclusion and achieved comparable outcomes in terms of skeletal and dental effects. Awad and Sadek [33] found that aligner mandibular advancement significantly improved Class II relationship, reduced overjet, and enhanced facial profile. These improvements were accompanied by better vertical control and improved upper lip positioning, resulting in a more balanced facial profile. Wu et al. [34] observed that aligner mandibular advancement reduced overjet effectively, with better control over lower incisors inclination compared to the Herbst and Twin Block appliances. Yue et al. [35] reported that both aligner mandibular advancement and the Twin Block expanded the airway and improved hyoid bone positioning. However, aligner mandibular advancement demonstrated greater effectiveness in increasing hypopharyngeal dimension. Hosseini et al. [36] found that aligner mandibular advancement excelled in controlling lower incisor proclination, while the Herbst appliance provided more substantial reduction in overbite. Lombardo et al. [37] revealed that both aligner mandibular advancement and the Twin Block significantly reduced overjet and ANB angle. While aligner mandibular advancement offered better vertical control, the Twin Block achieved slightly superior soft tissue outcomes, particularly in chin projection. Zhang et al. [38] demonstrated that aligner mandibular advancement and the Twin Block appliances promoted mandibular condylar growth and improved temporomandibular joint function. The Twin Block resulted in greater mandibular ramus elongation, whereas aligner mandibular advancement offered better comfort and patient compliance. Table 5 provides a summary of the key findings from the included studies.

**Table 5:** Main findings from the included studies CVM, cervical vertebral maturation

Authors and Year	Main Findings
Caruso et al., 2021 [30]	Both aligner mandibular advancement and the Twin Block effectively treated Class II malocclusion. Aligner mandibular advancement provided superior control over upper incisors, while the Twin Block showed a more pronounced mandibular elongation.
Ravera et al., 2021 [31]	Aligner mandibular advancement demonstrated significant dentoalveolar changes in prepubertal patients (CVM2), while pubertal patients (CVM3) showed more skeletal changes. Treatment effectively reduced overjet and ANB in both groups.
Zybutz et al., 2021 [32]	Patients preferred aligner mandibular advancement over the Twin Block due to better comfort, aesthetics, and reduced breakage. Both appliances showed comparable effectiveness in treating Class II malocclusion.
Awad and Sadek, 2022 [33]	Aligner mandibular advancement improved Class II relationships with significant skeletal and dental changes, including better control of vertical dimension and facial convexity improvement.
Wu et al., 2023 [34]	Aligner mandibular advancement was effective in reducing overjet and improving molar relationships, with better control over lower incisor inclination compared to the Herbst and Twin Block appliances. Skeletal changes were minimal compared to dental effects.
Yue et al., 2023 [35]	Both aligner mandibular advancement and the Twin Block expanded the airway and improved hyoid bone positioning. However, aligner mandibular advancement demonstrated better effectiveness in improving the hypopharynx area.
Hosseini et al., 2024 [36]	Both aligner mandibular advancement and the Herbst achieved Class II correction with significant skeletal and dental effects. Aligner mandibular advancement offered better control over mandibular incisor proclination, while the Herbst provided more significant overbite reduction.
Lombardo et al., 2024 [37]	Both aligner mandibular advancement and the Twin Block reduced overjet and ANB. The Twin Block showed slightly better chin projection improvements, while aligner mandibular advancement ensured better vertical control.
Zhang et al., 2024 [38]	Aligner mandibular advancement and the Twin Block promoted mandibular condyle growth and improved temporomandibular joint function. The Twin Block showed greater mandibular ramus elongation, while aligner mandibular advancement was more comfortable.

Discussion

Most of the studies included in this systematic review, along with some for which full texts were inaccessible, have been published within the past four years. This trend reflects a growing interest in understanding the effectiveness of aligner mandibular advancement for the treatment of Class II malocclusions in pediatric patients. Although several systematic reviews on clear aligners exist, their primary focus has often been on broader treatment outcomes, such as general efficiency or specific orthodontic mechanics. To the best of our knowledge, no systematic review had specifically addressed the aligner mandibular advancement in achieving mandibular correction and its associated treatment effects. Thus, this systematic review sought to fill that gap by providing a more focused, precise, and contemporary analysis of the effectiveness of aligner mandibular advancement in growing pediatric patients.

Skeletal and Dental Effects

The existing literature indicates that dental corrections achieved with the Herbst or Twin Block appliances account for 40% to 65% of the overall outcome achieved [39-41]. However, the question of whether functional appliances significantly enhance mandibular skeletal growth remains controversial, with studies yielding mixed results [42-45]. Caruso et al. [30] and Lombardo et al. [37] reported effective reductions in overjet and ANB, with aligner mandibular advancement offering superior control over upper incisor angulation compared to the Twin Block, which exhibited more pronounced mandibular elongation. This aligns with previous findings in the literature, where clear aligners demonstrated clinical effectiveness in managing upper incisor inclination during upper molar distalization [46]. Similarly, Awad and Sadek [33] highlighted reductions in overjet accompanied by enhanced facial convexity and vertical dimension control.

Growth stage was shown to influence outcomes. Ravera et al. [31] observed that prepubertal patients (CVM2) experienced dentoalveolar changes, while pubertal patients (CVM3) exhibited skeletal improvements. Wu et al. [34] supported these findings, demonstrating that aligner mandibular advancement provided better control of lower incisor angulation while achieving comparable reductions in overjet relative to the Herbst and Twin Block appliances.

Soft Tissue Effects

Hosseini et al. [36] demonstrated that aligner mandibular advancement significantly enhanced soft tissue profile by improving facial harmony and controlling lower incisor proclination. Similarly, Awad and Sadek [33] reported notable improvements in facial convexity and upper lip positioning, resulting in a more balanced and aesthetically pleasing profile. This finding is consistent with Hourfar et al. [47] who reported significant upper lip retrusion following treatment with the Herbst appliance. In comparison, the Twin Block appliance, as reported by Lombardo et al. [37], achieved greater advancement of the pogonion, enhancing chin projection more effectively than aligner mandibular advancement. Similarly, Shahamfar et al. [48] found that a modified Twin Block appliance effectively reduced facial convexity, corrected lip incompetence, and diminished upper lip protrusion.

Airway and Respiratory Effects

Yue et al. [35] reported that both aligner mandibular advancement and the Twin Block expanded the upper airway, with aligner mandibular advancement achieving superior improvements in hypopharyngeal dimension. This finding aligns with evidence in the literature [49-51]. Ozkoylu et al. [49] emphasized the respiratory benefits of the monoblock mandibular advancement device, highlighting its role in enhancing airway function. Adding to this, a systematic review by Serra-Torres et al. [50] revealed that mandibular advancement devices significantly reduced the frequency of apnea and hypopnea episodes, improved oxygen saturation levels, and alleviated snoring. Furthermore, Shi et al. [51] observed that mandibular advancement devices, whether designed with limited or free vertical opening, showed comparable efficacy in improving respiratory parameters and upper airway dimensions in patients with obstructive sleep apnea.

Comparison With Conventional Functional Appliances

Both aligner mandibular advancement and the Twin Block effectively corrected Class II malocclusion as evidenced by Caruso et al. [30] and Lombardo et al. [37]. However, aligner mandibular advancement consistently provided better control of upper and lower incisor angulation, which is a critical factor in achieving stable results. In contrast, the Twin Block demonstrated slightly greater mandibular elongation and improved chin projection, emphasizing its strength in enhancing skeletal correction. It is worth mentioning that both the Twin Block and mandibular advancement aligner operate on a similar mechanism, using inclined planes to guide the mandible into a forward position. When compared to the Herbst appliance, aligner mandibular advancement excelled in controlling lower incisor proclination as highlighted by Hosseini et al. [36]. However, the Herbst appliance was more effective in reducing overbite and managing vertical discrepancies, making it a more suitable option for patients requiring significant vertical correction.

Patient Comfort and Acceptance

Aligner mandibular advancement consistently outperformed traditional appliances in patient comfort and acceptance. Zybutz et al. [32] found that patients favored aligner mandibular advancement over the Twin Block due to its comfort, aesthetic appeal, and reduced appliance breakage. Zhang et al. [38] similarly highlighted aligner mandibular advancement's greater comfort and patient satisfaction while achieving comparable temporomandibular joint remodeling outcomes. These findings align with concerns raised by another study, which found that the size and noticeable color of the Twin Block appliance posed significant challenges to patient compliance [52]. Patients expressed a desire for a more discreet and less conspicuous device, as the high visibility of the Twin Block continued to cause social discomfort, even after a period of adjustment.

Limitations

The evidence included in this review had several limitations that may affect the interpretation of the results. First, most of the included studies were retrospective in design [30,33-38], which inherently carries a higher risk of selection and reporting biases than prospective studies. Additionally, no studies employed randomization, and confounding factors, such as compliance and growth, were not consistently accounted for, which may have influenced the outcomes. Another limitation was the heterogeneity in treatment protocols, patient demographics, and assessment methods. While most studies used lateral cephalometric radiographs for outcome measurement, some incorporated CBCT [35,38], leading to variability in data collection. Furthermore, sample sizes varied considerably, limiting the generalizability of some findings. Treatment outcomes for aligner mandibular advancement were often compared to different functional appliances, making direct comparisons across studies challenging. Soft tissue outcomes were reported in limited studies, highlighting an evidence gap in this area.

While rigorous methods were employed in conducting this systematic review, certain limitations must be acknowledged. Only studies published in English were included, which may have introduced language bias and excluded relevant studies published in other languages. Limited studies provided detailed information on the extent of compliance with treatment protocols. Additionally, due to the heterogeneity of study designs, protocols, and outcome measures, a quantitative meta-analysis could not be performed. This limited the ability to draw statistically robust conclusions.

Implications of the Results for Practice and Future Research

The findings of this review provide valuable insights into the effectiveness of aligner mandibular advancement in the treatment of Class II malocclusion. Clinically, the results support its use as a viable alternative to conventional functional appliances, particularly for patients who prioritize aesthetics and comfort. However, clinicians should be mindful of the current evidence limitations, including variability in treatment outcomes and patient-specific factors such as age and compliance. The growing body of evidence emphasizes the need for standardized treatment guidelines and uniform outcome assessment protocols in orthodontics. High-quality randomized controlled trials are essential to overcome the limitations associated with retrospective and non-randomized studies. Additionally, long-term follow-up is necessary to assess the stability of treatment outcomes, especially in growing patients.

Further research should also explore soft tissue effects, patient-reported outcomes, and the cost-effectiveness of aligner-based interventions. Strengthening the evidence base in these areas will help refine and optimize treatment strategies for Class II malocclusion in clinical practice.

## Conclusions

This systematic review suggests that clear aligner mandibular advancement appears to be an effective treatment option for managing Class II malocclusion in growing pediatric patients. The correction is achieved through a combination of dental and skeletal effects, with the appliance showing a significant advantage in maintaining lower incisor inclination compared to conventional functional appliances. Furthermore, aligner mandibular advancement was preferred by patients due to its superior comfort, enhanced aesthetics, and reduced risk of appliance breakage.

Despite these promising outcomes, the current evidence is constrained by significant methodological limitations, including the absence of randomized controlled trials, small sample size, and variability in study designs. Future research should prioritize addressing these gaps by employing robust study designs, larger patient populations, and standardized treatment protocols. This will help establish more reliable evidence to inform clinical practice and optimize treatment outcomes.
